# Isolation and Characterization of a New Thermoalkalophilic Lipase from Soil Bacteria

**Published:** 2015

**Authors:** Mohammad Rabbani, Fatemeh Shafiee, Zahra Shayegh, Hamid MirMohammadSadeghi, Ziaedin Samsam Shariat, Zahra Etemadifar, Fatemeh Moazen

**Affiliations:** a *Department of Pharmaceutical Biotechnology, School of Pharmacy and Pharmaceutical Sciences, Isfahan University of Medical Sciences, Isfahan, Iran.*; b *Department of Biochemistry, School of Pharmacy and Pharmaceutical Sciences, Isfahan University of Medical Sciences, Isfahan, Iran.*; c *Department of Biology, School of Basic Sciences, University of Isfahan, Isfahan, Iran.*

**Keywords:** Thermoalkalophile, Lipase, *Bacillus subtilis*, Soil

## Abstract

Lipases are diversified enzymes in their properties and substrate specificity, which make them attractive tools for various industrial applications. In this study, an alkalinethermostable lipase producing bacteria were isolated from soil of different regions of Isfahan province (Iran) and its lipase was purified by ammonium sulfate precipitation and ion exchange chromatography. To select a thermoalkalophil lipase producing bacterium, Rhodamine B and Horikoshi media were used and the strain that can grow at 45*°C*was selected. The isolated strain was identified using microbial and biochemical tests.

One strain showed an orange colored zone on plate and grew on Horikoshi plate. Microbial and biochemical tests showed that the isolated strain was *Bacillus subtilis*, a Gram positive rod. In PCR, an expected band was obtained with about 371 bp. The activity of the purified lipase was 10.2 folds that of the standard enzyme using ammonium sulfate precipitation and ion exchange chromatography. The molecular weight of lipase determined by SDS-PAGE electrophoresis, was 21 and 35 KDa.

Existence of two bands in SDS-PAGE electrophoresis and low amount of obtained purified enzyme highlights the necessity of optimization of purification and concentrating process.

## Introduction

Lipases are a class of enzymes that facilitate the hydrolysis of mono, di and triglycerides into free fatty acids and glycerol (1, 2). These enzymes exist widely in nature in several sources, such as animals, plants, fungi, bacteria and yeasts (3). Among these sources, microbial lipase properties such as, unique stability in organic solvents, lack of need for cofactor, specific responses to a wide variety of substrates and enantioselectivity are more important compared to other sources for biotechnological processes (4). In addition, having a higher thermal stability and activity in different pH from neutral to basic are other desirable characteristics of these microbial resources (5). 

Considering the widespread use of these enzymes in different industries, nowadays many efforts are made to find enzymes with specific properties for optimal efficiency of industrial processes (5). Due to the high melting temperature of the lipase substrate, often these processes are carried out at 40 *°C *and higher. The lipase that is used in these reactions must be able to withstand temperatures of about 50 *°C (*3*)*. In general, thermostable enzymes are obtained from thermophilic or mesophilic microorganisms (6, 7). Thermophilic microorganisms that are important sources for this enzyme, are normally isolated from the soil of areas with special temperature conditions (8). Among the available sources, only certain species have acceptable biosynthetic capabilities for use in organic reactions. These species include* Achromobacter, Arthrobacter, Bacillus* and *Pseudomonas* (9).

Interesting features of *Bacillus subtilis* such as the ability to secrete homologous and heterologous proteins in significant amounts to medium culture, classification as a safe bacterium by the FDA, the ability of endospore production, the ability to live in different conditions of heat and drought and the ability to produce commercially important enzymes make this microorganism as an important source for the production of commercial enzymes (10). The present study was aimed to isolate and characterize the lipase producing bacteria from soil samples collected from different regions of Isfahan, Iran.

## Experimental

*Materials*

Nutrient agar, nutrient broth, Simmon citrate agar, agar, Voges-Proskauer medium, acrylamide, bisacrylamid, TEMED, ammonium persulfate, EDTA, bromophenol blue, glacial acetic acid, and NaCl were purchased from Merck (Germany). Ethidium bromide and loading buffer were purchased from Cinnagen(Tehran, Iran) and DNA ladder from Fermentas (Poland). Oligonucleotide primers, deoxynucleotides and Taq polymerase were purchased from Bioron (Germany).

*Collection and preparation of soil samples*

Soil samples were collected from different areas of Iran, were placed at room temperature and in the open air for 48 hours to kill all the vegetative forms and stimulate the spore generation (11). The samples (10 g) were then heated to 80*°C and dissolved in distilled water.* Soil solution was allowed to settle and then the supernatant was serially diluted from 10^-1^,10^-2^, 10^-3^ and 10^-4^. 

*Microbial and biochemical tests*

 Microbial and biochemical tests were also used for identification of isolated lipase positive colonies. According to the Bergey’s manual of systemic bacteriology, these tests were selected and were performed in triplicate (12). The tests included: Gram staining reaction, spore position and shape, aerobic or anaerobic growth, Vogues-Proskauer test, oxidase, catalase, citrate consuming, nitrate reductase and lecitinase reaction. The cell morphology was examined by light microscopy and biochemical characteristics were investigated at room temperature and 37 °C.

Growth under anaerobic conditions was checked by inoculating a trypton soy broth with the isolated strains and incubating in an anaerobic jar supplemented with a gas pack strip type A for several days. 

*Lipase activity assays*

The lipolytic activity of strains was detected on TW agar plates containing 1% Tributyrin and 1% Tween 80 (pH 8). Lipase production was detected by observing clear zones around isolated colonies (12). Colonies with a clear halo were selected in Rhodamine B medium in 350 nm wavelength (13). Colonies which showed orange fluorescence under UV irradiation indicated true lipase activity and non-lipolytic bacteria formed pink colonies. To identifyalkalophil species, Horikushi medium was used; growth in this culture medium indicated that microorganisms are alkalophil (14). To screen the alkalophil species, selected colonies were incubated at 30, 35, 40, 45, and 50 *°C* and growth was assessed after 24-48 hours.

Lipase activity was also determined by colorimetric assay based on amount of the released p-nitrophenol from enzymatic reaction on p-nitrophenoldecanoate (11). Activity measurements were routinely performed in Tris buffer (50 mM, pH 8) containing 10 µL 4-nitrophenyl decanoate(50 mM) as the substrate, 25 µL gum Arabic (10%), and 50 µLsodium taurocholate (20 mM), final concentrations. The reaction mixture was incubated in water bath at 37 °C for 10 minutes, and then the reaction was started by addition of 25 µL of enzyme solution and continued for additional 5 minutes at 37 °C. Activity was measured spectrophotometrically at 400 nm using the BioTek Powerwave XS reader. One unit was defined as the amount of enzyme that released 1 µM 4-nitrophenol per minute per milligram protein. To measure the enzymatic activity, it is necessary to plot the standard curve (absorbance versus concentration).To assess the Specific enzyme activity the following equation was used:

Specific enzyme activity µmolmg=activity (µmol/mg)protein (mgml)

*Identification of isolated bacteria by PCR*

PCR reaction was performed byTaq polymerase (5U/mL), dNTP (100 µM), specific primers (25 µM) and X-PCR buffer (5 µM).The specific primers used included: 

Sense: 5-ATGGTTCACGGTATTGGAGG-3

Antisense: 5-CTGCTGTAAATGGATGTGA-3

**Figure 1:**
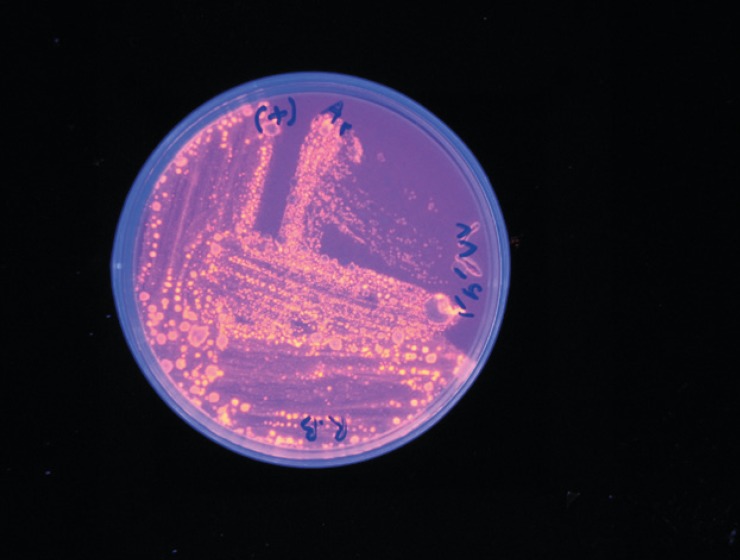
large colonies from lecitinase medium were transferred to Rhodamine B medium. Colonies with a clear halo in 302 nm wavelength were selected in this medium

The PCR conditions were as follows: one initial denaturation step at 94 °C for 5 minutes, 35 cycles at 94 °C for 1 minute, gradient temperature (60.2, 56.6, 52, 48.6, 46.3 and 35 *°C)* for 2 minutes, extension at 72 °C for 3 minutes, and a final extension for 5 minutes. The PCR product was run to agarose gel (0.7 %w/v) electrophoresis and the gel containing DNA was observed at 302 nm wavelength (15) (Figure1).

*Partial purification of lipase*

*Ammonium sulfate precipitation*

Solid ammonium sulfate was added to the cell free supernatant at 30% saturation and allowed to stand for 3 h. The precipitate was separated by centrifugation and solid ammonium sulfate was added to the supernatant at 80% saturation. The precipitates were resuspended in a minimal amount of Tris buffer (pH 10.9) at 4 *°C* and the solution was dialyzed against the same buffer to remove residual ammonium sulfate.

*Ion exchange chromatography*

Ten mL of prepared Q-Sepharose fast flow resin(an anion exchanger) was seeded to column and after the precipitation of resin, the column was eluted by Tris buffer (pH 10.9). The dialyzed sample was added to the column and the washing was performed with 50 mMTris buffer seeded to the column. Finally, the attached proteins (specially lipase enzyme) was eluted by Tris buffer (pH=10.9) with increasing concentrations of NaCl (0.1-0.5 mM) for replacing the Cl^-^ with lipase and separation it from positive charged resin. After assaying the fractions for lipase activity, fractions showing highest activity were pooled together and stored at -20 °C.

*SDS-PAGE gel electrophoresis*

**Figure 2:**
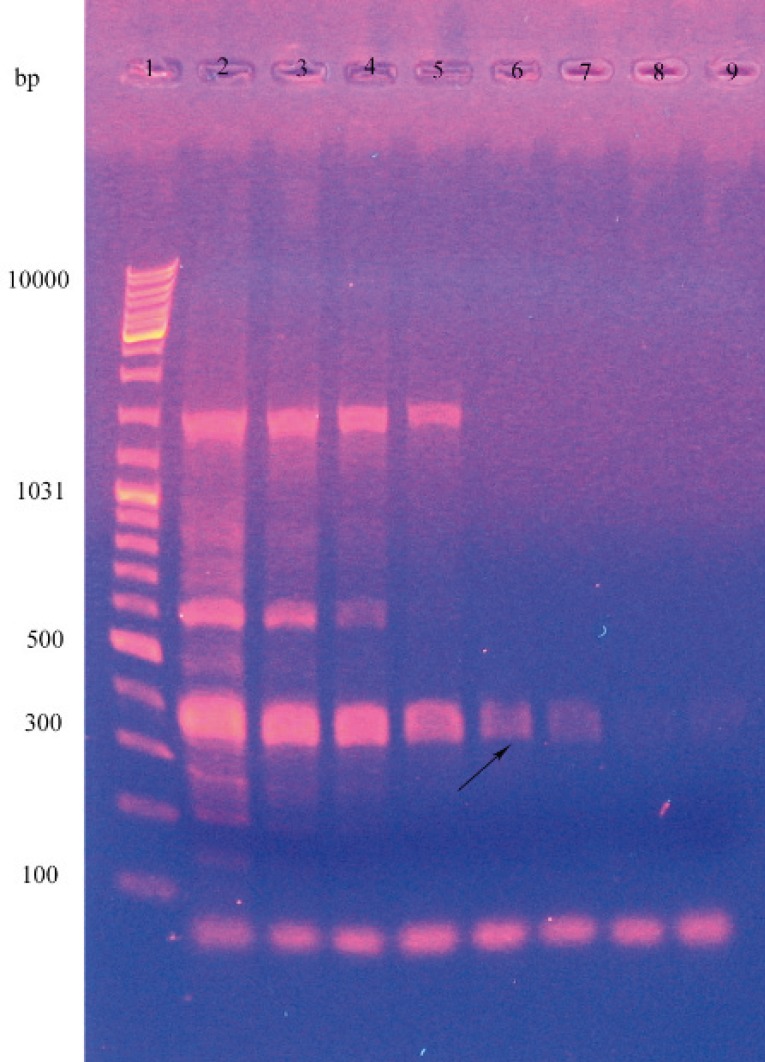
PCR amplification of the *B. subtilis* lipase A gene. In this experiment the DNA templates used were as follows: lane 1: leader, lane 2 to 9: temperature gradients. (2: 45, 3:46.3, 4: 48.6, 5: 52, 6:56.6, 7: 60.2, 8: 62.5 and 9: 64).

Crude cell extracts were analysed by SDS-PAGE on a 12% stacking gel and 10% separating gel. Gels were stained for protein detection by Coomassie brilliant blue G-250. The gel was shacked at room temperature for 1 hour. Then the gel put in bleaching solution to identify protein bands. For determination the molecular weight, markers with particular molecular weight were seeded to wells closed to the sample and the molecular weight of lipase was determined based on the markers molecular weight (16) (Figure 2).

## Results

The lipase producing bacteria were isolated from different soil samples. Among 64 colonies with the ability of production of colored halo on lecitinase medium, four colonies created orange halo in 350 nm wavelength (Rhodamine positive strains) (Figure 1).After culturing the bacteria in the Horikoshi medium, only one colony was able to grow on this environment (alkalophil strain). After incubation the alkalophil colony in different temperatures (30, 35, 40, 45, and 50 *°*C), the most suitable temperature was determined to be 45* °*C.

All the isolated strains with positive Rhodamine test were characterized first by conventional biochemical techniques and were further characterized by PCR amplification. Based on various microbial and biochemical testes, it was showed that the bacterium was a Gram positive strain. Existence of rod endospores indicated that the isolated strain was a* Bacillus* specie, and the results of biochemical tests, (Catalase positive, Lecitinase negative, Citrate positive, Anaerobic growth negative, Casein positive, Starch positive, Nitrate reduction positive, Sugar fermentation positive and Voges-Proskauer positive) showed that the isolated strain was *Bacillus subtilis*.

For precise determination of the species, the specific primers were used to identify the lip A gene, and consequently a band was observed at about 371 bp on gel electrophoresis. Temperature gradiants (45,46.3,48.6,52, 56.6, 60.2, 62.5 and 64*°*C) for amplifying the lipase gene, produced the lipase fragment in all of them but by increasing the temperature the severity of band and consequently the amount of amplified gene was decreased (Figure 2).

**Figure 3:**
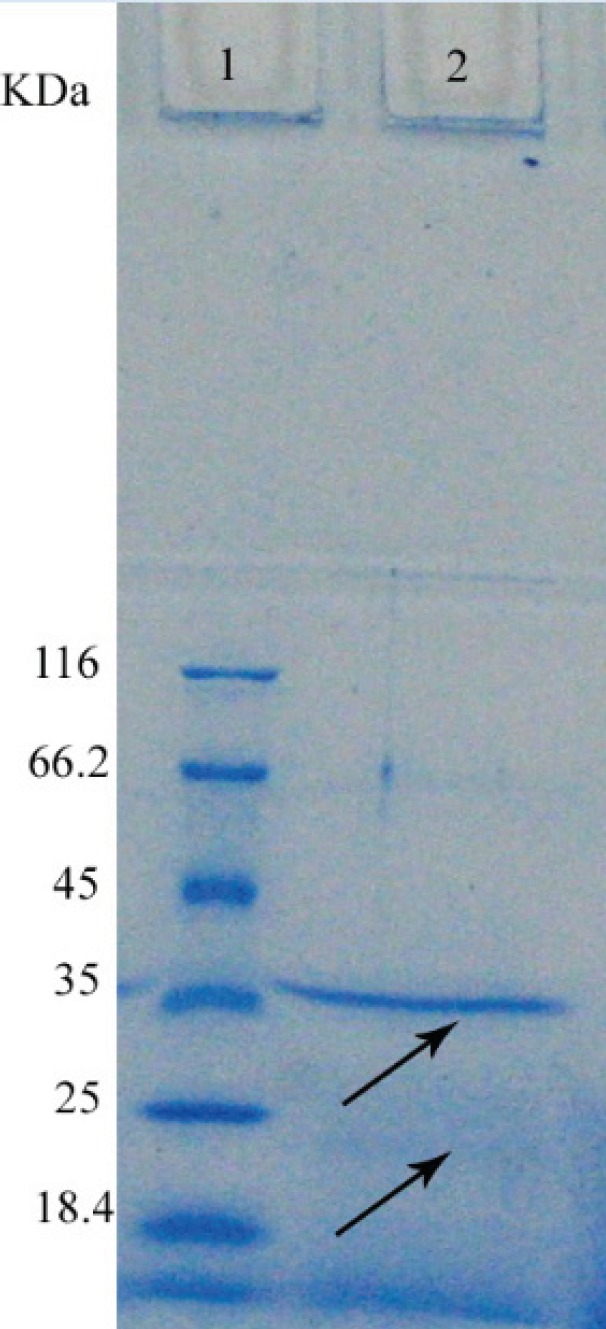
SDS-PAGE analysis of purified lipase in a 12% polyacrylamide gel. In this experiment the protein templates used were as follows Lane 1: the standard molecular weight marker (kDa), lane 2: purified sample with 31(kDa).

For purification of the enzyme by ion exchange chromatography, considering the pI of enzyme is 9.9, it had negative charge and was attached to resin in pH of 10.9. This purified protein was quantified using Bradford assay (0.56 mg) and used for determining the enzyme activity. Specific enzyme activity was found to be 2.1 U/mg that was 10.2 folds of the standard enzyme (0.21 U/mg). SDS-PAGE electrophoresis revealed a band of 35 KDa (Figure 3).

## Discussion

The aim of this study was to isolate and characterize the lipase producing bacteria from soil samples collected from different regions of Isfahan, Iran.

By using the specific primers were used to identify the lip A gene, a band was observed at about 371 bp on gel electrophoresis and this Corroborated the lipase gene (8). But in agarose gel electrophoresis two other bands was observed that with increasing the temperature they was diminished. This means that PCR amplified nonspecific fragments in addition of lipase gene. However because the primers are specific for the lipase gene, this fragment is more and by increasing the temperature, primer nonspecifically bonding and consequently amplifying the non target sequences were diminished. 

Para-nitrophenol decanoate is used routinely for Measurement the activity of different enzymes, but in this study the activity of lipase was determined after the purification of it and therefore the activity measured is attributed to our lipase.

On the SDS-PAGE electrophoresis only one band was observed; that was 35KDa. Lipase in *Bacillus subtilis*is about 19-20 KDa (17) and in Bacillus and Pseudomonas tend to aggregate because of many hydrophobic amino acids in its structure (18). This affinity is responsible for observation of 24 and 48 KDa bands for the lipase of *Bacillus thermocatenulatus*, although it is expectable for the molecular weight of this enzyme to be 16 KDa (8). Thus the band in 35 KDa is probably due to the aggregation of the lipase. High affinity of this lipase causes aggregation and decrease of the final product.

Performing of western blotting method for the lipase in not possible because of the absence of specific antibody against this protein and SDS-PAGE can't prove the presence of lipase carefully.

The purified enzyme in this project was 10.15 times more active compared to the standard lipase. This activity is less compared to other studies that used other methods of concentrating and purification on *Bacillus subtiliis*. In Ahmad EH*, et al.* study that used ammonium sulfate and phenyl sepharose to purify lipase EH37, the activity of purified enzyme was 17.8 folds that of the standard enzyme (10). In another project, the activity of the enzyme was obtained as 67%, using precipitation by methanol and purification by ammonium sulfate for Bacillus thermocatenulatus (18). Low activity and recovery in the purification process indicate the necessity of optimization of purification and concentrating process.

In conclusion, the separated enzyme in this project can be used as a thermostable, resistant to severe pH. However, to obtain a better result, it is necessary to optimize the purification and concentrating process.
